# Central Aortic Cannulation in Minimally Invasive Cardiac Surgery via Right Thoracotomy: A Single-Center Retrospective Comparison

**DOI:** 10.3390/jcm15062383

**Published:** 2026-03-20

**Authors:** Tayfun Özdem, Tuna Demirkıran, Mesut Akyol, Işıl Taşöz Özdaş, Furkan Burak Akyol, Yiğit Tokgöz, Veli Can Özdemir, Emre Kubat, Gökhan Arslan, Murat Kadan, Kubilay Karabacak

**Affiliations:** 1 Department of Cardiovascular Surgery, Gülhane Training and Research Hospital, Ministry of Health, 06010 Ankara, Turkey; drtunademirkiran@gmail.com (T.D.);; 2 Department of Biostatistics, Ankara Yıldırım Beyazıt University Medical Faculty, 06010 Ankara, Turkey; 3 Department of Cardiovascular Surgery, Gülhane Training and Research Hospital, University of Health Sciences, 06010 Ankara, Turkey; 4 Department of Cardiovascular Surgery, İskenderun Gelişim Hospital, 31200 Hatay, Turkey; ekubat@gmail.com; 5 Department of Cardiovascular Surgery, Memorial Ankara Hospital, 06010 Ankara, Turkey

**Keywords:** central aortic cannulation, femoral arterial cannulation, minimally invasive cardiac, surgery, right thoracotomy, cardiopulmonary bypass, arterial cannulation

## Abstract

**Background/Objectives:** Minimally invasive cardiac surgery via right thoracotomy commonly uses femoral arterial cannulation for cardiopulmonary bypass (CPB), which requires an additional groin incision and may be associated with access-related complications. Central aortic cannulation through the same thoracotomy allows antegrade perfusion without an extra incision. This study compared central aortic and femoral arterial cannulation strategies in minimally invasive cardiac surgery via right thoracotomy. **Methods**: This retrospective, single-center study included 139 consecutive patients undergoing minimally invasive right thoracotomy cardiac surgery with CPB between February 2021 and June 2023. Patients were grouped according to arterial cannulation strategy: central aortic cannulation (*n* = 93) and femoral arterial cannulation (*n* = 46). Demographic characteristics, operative variables, transfusion requirements, biochemical parameters, and early postoperative clinical outcomes were compared between the groups. **Results**: Baseline demographic characteristics differed between groups (age, height, body surface area, and sex distribution), and these differences should be considered potential confounders when interpreting outcome comparisons. Central cannulation was more frequently used in women (74.2% vs. 45.7%, *p* = 0.001). Patients in the femoral group were older (median 61.0 vs. 54.0 years, *p* = 0.004), taller (1.65 ± 0.10 vs. 1.59 ± 0.09 m, *p* < 0.001), and had a slightly higher body surface area (*p* = 0.043). Cross-clamp and CPB durations were longer in the femoral group (cross-clamp: 90.0 vs. 70.5 min, *p* = 0.015; CPB: 137.0 vs. 114.0 min, *p* = 0.003). Lymphatic leakage occurred in three patients in the femoral group (6.5% vs. 0%, *p* = 0.009). No significant differences were observed between groups for mortality, intensive care unit stay, or neurological events. **Conclusions**: Central aortic cannulation via right thoracotomy is a feasible alternative to femoral arterial cannulation, enabling antegrade perfusion without a groin incision, reducing operative time, and potentially decreasing access-related complications such as lymphatic leakage, while yielding comparable early clinical outcomes. These findings should be interpreted cautiously given the retrospective design and baseline differences between groups.

## 1. Introduction

Minimally invasive cardiac surgery (MICS) via right thoracotomy has become an established alternative to median sternotomy for selected valve and congenital heart procedures. In addition to its cosmetic benefits, reduced surgical trauma has been associated with lower transfusion requirements, shorter hospital stay, and faster postoperative recovery [1]. Several contemporary large series have demonstrated that minimally invasive cardiac surgery is associated with reduced transfusion requirements, shorter intensive care unit stay, and earlier hospital discharge compared with conventional sternotomy [2,3,4,5]. Right thoracotomy–based minimally invasive surgery has proven to be an effective and reproducible approach with reasonable operative times and low morbidity rates, thereby extending its advantages beyond cosmetic outcomes alone [6].

However, cardiopulmonary bypass (CPB) in MICS frequently relies on peripheral arterial cannulation, most commonly via the femoral artery, which necessitates an additional groin incision [7,8,9]. Femoral arterial cannulation (FAC) can be achieved using either surgical or percutaneous techniques. Although percutaneous approaches may reduce local wound morbidity, they are associated with additional device-related costs and do not eliminate the risk of vascular complications. Furthermore, retrograde arterial perfusion has been associated with neurological and aortic complications in selected patient populations, particularly in the presence of peripheral vascular disease or aortic atherosclerosis; however, the available evidence remains mixed, and the potential influence of patient selection on the actual risk should be considered [7,10,11].

In addition, vascular and local complications associated with femoral cannulation may negatively affect clinical outcomes, especially in elderly patients and those with comorbidities [10]. Large patient series comparing antegrade and retrograde perfusion strategies have demonstrated that the direction of perfusion may play a decisive role in clinical outcomes [11].

In this context, central aortic cannulation (CAC), performed through the same thoracotomy incision, has emerged as an alternative strategy that enables antegrade perfusion without requiring an additional groin incision. Several studies have demonstrated that CAC can be applied effectively in minimally invasive cardiac surgery [12,13,14]. Additional evidence from different clinical contexts further supports the use of CAC in minimally invasive procedures [15,16]. Studies evaluating cannulation strategies in related clinical scenarios have also provided important insights, suggesting that central cannulation may be associated with fewer limb-related vascular complications compared with peripheral approaches, while similar postoperative outcomes have been reported between central and peripheral strategies in selected settings [17,18]. Comparable early outcomes have also been described with alternative arterial strategies in minimally invasive mitral valve surgery [2]. Furthermore, both central and peripheral cannulation techniques have been emphasized to have context-dependent advantages and limitations [19]. Nevertheless, data directly comparing central aortic cannulation with femoral arterial cannulation in right thoracotomy minimally invasive cardiac surgery remain limited.

The present study aims to compare central aortic and femoral arterial cannulation for cardiopulmonary bypass in patients undergoing minimally invasive cardiac surgery via right thoracotomy, with particular focus on operative times, perioperative complications, and early postoperative clinical outcomes.

## 2. Materials and Methods

### 2.1. Ethics Approval and Informed Consent

The study protocol was reviewed and approved by the institutional ethics committee of Gülhane Training and Research Hospital (approval number: 2023/288, date: 22 November 2023). The study was conducted in accordance with the principles of the Declaration of Helsinki and its later amendments. Owing to the study’s retrospective nature and the use of anonymized patient data, the ethics committee waived the requirement for written informed consent.

### 2.2. Study Design and Patient Population

This retrospective, single-center observational study included 139 consecutive patients treated between February 2021 and June 2023 who underwent MICS via right thoracotomy with CPB. During the early phase of the study period, femoral arterial cannulation was routinely used for cardiopulmonary bypass. Beginning in March 2022, central aortic cannulation became the preferred institutional strategy, reflecting a transition in routine cannulation practice. Femoral arterial cannulation was subsequently reserved for selected cases in which central cannulation was considered unsuitable, particularly in the presence of significant ascending aortic calcification.

Patients were stratified according to the arterial cannulation strategy used during CPB: central aortic or femoral arterial cannulation.

For procedure-specific subgroup analyses, patients were further categorized by surgical procedure type: atrial septal defect repair, mitral valve replacement with tricuspid valve repair, isolated mitral valve replacement, and other minimally invasive cardiac procedures. The latter group included isolated tricuspid valve replacement, isolated aortic valve replacement, combined aortic and mitral valve replacement, intracardiac mass excision, combined aortic–mitral–tricuspid valve replacement, and combined mitral–tricuspid valve surgery with atrial septal defect repair.

Baseline demographic characteristics, comorbidities, and operative variables—including aortic cross-clamp time and CPB duration—were recorded. Transfusion requirements and early postoperative clinical outcomes were evaluated. Postoperative outcomes included intensive care unit stay, hospital length of stay, and procedure-related complications such as vascular embolic events, neurological events, deep vein thrombosis, lymphatic leakage, and in-hospital mortality.

Neurological events were defined as postoperative stroke or transient ischemic attack confirmed by neurological evaluation. Vascular embolic events were defined as clinically evident peripheral embolism confirmed by imaging or requiring intervention. Deep vein thrombosis was defined as thrombosis confirmed by duplex ultrasonography. Lymphatic leakage was defined as persistent lymphatic drainage from the groin wound requiring prolonged drainage or additional intervention.

Given the relatively small number of patients within individual procedure categories, these subgroup analyses were considered exploratory and were not powered for definitive comparisons; therefore, the findings should be interpreted with caution.

### 2.3. Laboratory Assessment

Biochemical parameters, including urea, creatinine, aspartate aminotransferase, alanine aminotransferase, neutrophil count, lymphocyte count, and platelet count, were assessed preoperatively and postoperatively.

### 2.4. Surgical Strategy

All procedures were performed through a right thoracotomy approach by a single experienced cardiovascular surgeon. To initiate central cannulation, U-sutures were placed on the ascending aorta as distally as feasible through the thoracotomy incision without extending the skin incision, using 2-0 pledget nonabsorbable polyester sutures in a cross configuration and prepared with snares. The central area was opened with an 11-blade scalpel, and an appropriately sized arterial cannula was inserted over a dilator. Cannula position and aortic anatomy were assessed intraoperatively under direct vision and, when necessary, with transesophageal echocardiographic guidance to confirm appropriate cannula tip location and exclude malposition or aortic wall injury. To prevent excessive advancement, the cannula was secured with a preplaced stabilization tape limiting insertion to approximately 2–3 cm. After tightening the snares, the dilator was withdrawn and CPB was initiated in the standard fashion.

**Figure 1:**
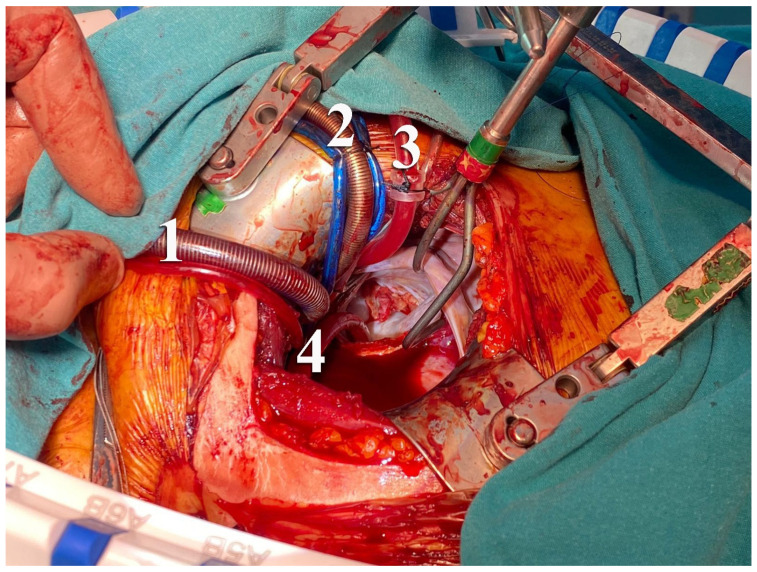
Central aortic cannulation during right thoracotomy. Intraoperative view demonstrating central aortic cannulation through a right thoracotomy approach. (1) Superior vena cava cannula, (2) central aortic arterial cannula, (3) aortic root cannula, and (4) pulmonary vent suction are shown. Central arterial cannulation was performed under direct vision without extension of the thoracotomy incision.

**Figure 2:**
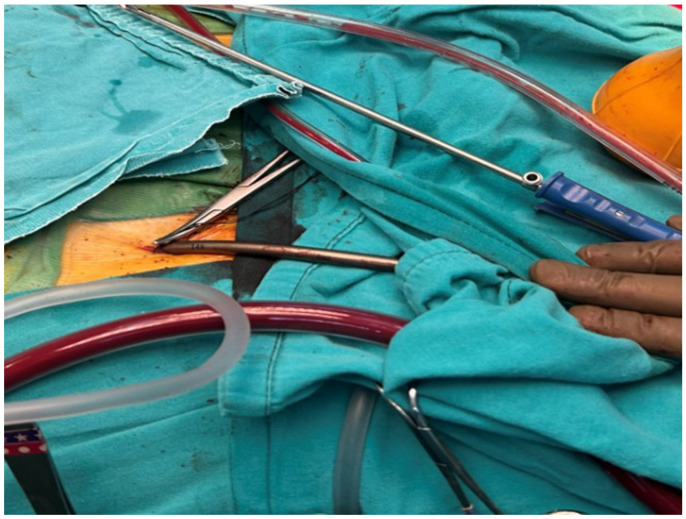
Percutaneous femoral venous cannulation. Intraoperative view of percutaneous femoral venous cannulation used for cardiopulmonary bypass during minimally invasive cardiac surgery. The venous cannula is inserted percutaneously through the femoral vein.

**Figure 3:**
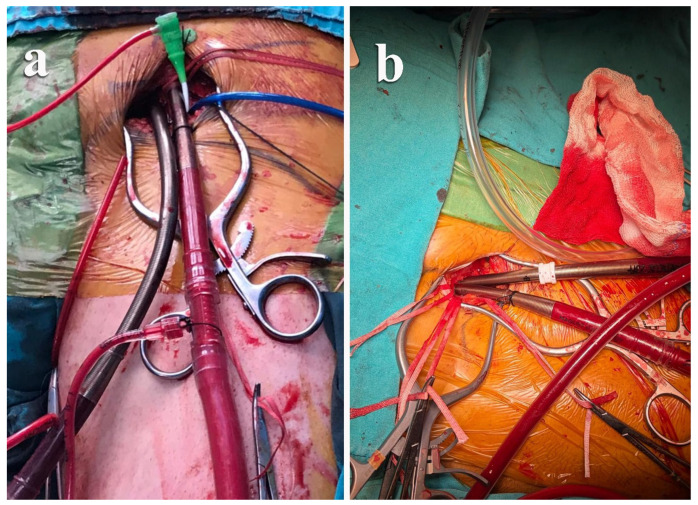
Surgical femoral arterial cannulation. Intraoperative views of surgically exposed femoral arterial and venous cannulation for cardiopulmonary bypass. (**a**) The femoral artery and vein were exposed through a standard groin incision and cannulated under direct vision. (**b**) Completed femoral arterial and venous cannulation setup during cardiopulmonary bypass. Distal perfusion cannulation was not routinely used.

In selected cases, FAC was preferred and performed via standard surgical exposure of the common femoral artery. Venous drainage strategy was applied according to institutional routine. The surgical exposure and cannulation techniques used for central aortic and femoral arterial cannulation during right thoracotomy are illustrated in Figure 1, Figure 2 and Figure 3.

### 2.5. Statistical Analysis

Statistical analyses were performed using IBM SPSS Statistics for Windows (version 22.0; IBM Corp., Armonk, NY, USA). Data distribution was assessed for normality using the Shapiro–Wilk test. Continuous variables are presented as mean ± standard deviation or median (interquartile range), as appropriate. Categorical variables are expressed as numbers and percentages.

Comparisons between independent groups were conducted using the independent samples *t*-test or the Mann–Whitney U test for continuous variables, and the χ^2^ test or Fisher’s exact test for categorical variables, as appropriate. Paired comparisons of preoperative and postoperative laboratory parameters were performed using the Wilcoxon signed-rank test. A two-sided *p*-value < 0.05 was considered statistically significant.

To account for baseline differences between groups, multivariable linear regression analyses were performed for operative time parameters, including cardiopulmonary bypass duration and aortic cross-clamp time as dependent variables. Cannulation strategy was included as the main independent variable, and age, sex, body surface area, and hypertension were entered as covariates based on their clinical relevance and baseline differences between groups.

## 3. Results

This retrospective single-center study included 139 patients (49 men [35.3%] and 90 women [64.7%]). Age ranged from 17 to 77 years, with a mean age of 55.06 ± 15.51 years. The most frequently performed procedures were isolated mitral valve replacement (*n* = 51, 36.7%) and atrial septal defect repair (*n* = 26, 18.7%). CAC was performed in 93 patients (66.9%) and FAC in 46 patients (33.1%).

### 3.1. Baseline Characteristics

Baseline characteristics according to arterial cannulation strategy are summarized in Table 1. CAC was more frequently used in women (74.2% vs. 45.7%, *p* = 0.001). Patients in the femoral cannulation group were older (median 61.0 vs. 54.0 years, *p* = 0.004) and taller (1.65 ± 0.10 vs. 1.59 ± 0.09 m, *p* < 0.001). Body surface area was slightly higher in the femoral cannulation group compared with the central cannulation group (*p* = 0.043), whereas hypertension was more prevalent in the central cannulation group (*p* = 0.013). No other baseline variables differed significantly between groups (all *p* > 0.05). Although the EuroSCORE distribution differed numerically between groups, the difference was not statistically significant (*p* = 0.102) and was therefore not included in further adjusted analyses.

In procedure-specific subgroup analyses, the proportion of women undergoing central cannulation was higher among patients who underwent isolated mitral valve replacement (*p* = 0.019). In all procedure categories other than isolated mitral valve replacement, patients who underwent femoral cannulation were older than those who underwent central cannulation (*p* < 0.05). Among patients undergoing isolated mitral valve replacement, height was greater in the femoral cannulation group (*p* = 0.006). Other demographic variables did not differ by procedure type and cannulation strategy (all *p* > 0.05) (see Supplementary Table S1).

### 3.2. Operative and Early Postoperative Outcomes

Operative and early postoperative outcomes by cannulation strategy are presented in Table 2. Aortic cross-clamp time and CPB duration were significantly longer in the femoral cannulation group compared with the central cannulation group (cross-clamp: median 90.0 vs. 70.5 min, *p* = 0.015; CPB: median 137.0 vs. 114.0 min, *p* = 0.003). The number of platelet units transfused was higher in the central cannulation group (*p* = 0.020). No significant differences were observed in intensive care unit stay, hospital length of stay, red blood cell transfusion, fresh frozen plasma transfusion, vascular embolic events, or in-hospital mortality (all *p* > 0.05). Lymphatic leakage occurred in three patients in the femoral cannulation group (6.5% vs. 0%, *p* = 0.009). No significant differences were observed between groups for neurological events. Although the median platelet transfusion values were identical between groups (median = 4 units), the overall distribution differed, with higher extreme values observed in the central cannulation group, resulting in a significant Mann–Whitney U test.

In procedure-specific analyses, patients undergoing mitral valve replacement with tricuspid repair had longer aortic cross-clamp and CPB durations when femoral cannulation was used (*p* < 0.001). No other procedure categories showed significant differences in operative or early postoperative variables between cannulation strategies (all *p* > 0.05) (see Supplementary Table S2).

To account for baseline differences between groups, multivariable linear regression analyses were performed including cannulation strategy, age, sex, body surface area, and hypertension as covariates. For cardiopulmonary bypass duration, the overall model was statistically significant (R^2^ = 0.205, adjusted R^2^ = 0.175, *p* < 0.001). Cannulation strategy remained independently associated with CPB duration (B = 37.49, β = 0.343, *p* < 0.001). Age was also independently associated with longer CPB duration (B = 1.32 per year increase, β = 0.397, *p* < 0.001), whereas sex (*p* = 0.889), body surface area (*p* = 0.992), and hypertension (*p* = 0.262) were not significant predictors.

For aortic cross-clamp time, the multivariable regression model was not statistically significant overall (R^2^ = 0.047, adjusted R^2^ = 0.012, *p* = 0.258). Cannulation strategy was not independently associated with cross-clamp duration (B = 10.21, β = 0.129, *p* = 0.158). However, increasing age was associated with longer cross-clamp time (B = 0.49 per year increase, β = 0.204, *p* = 0.033), while sex (*p* = 0.615), body surface area (*p* = 0.785), and hypertension (*p* = 0.813) were not significant predictors.

### 3.3. Laboratory Parameters

Perioperative laboratory parameters are shown in Table 3. Between-group comparisons demonstrated no significant differences in urea, creatinine, aspartate aminotransferase, alanine aminotransferase, neutrophil count, lymphocyte count, or platelet count at the preoperative and postoperative time points (all *p* > 0.05). Procedure-specific subgroup comparisons of laboratory parameters are provided in Supplementary Table S3.

## 4. Discussion

This retrospective, single-center study compared two arterial cannulation strategies—CAC and FAC—for establishing CPB in MICS performed via right thoracotomy. The most important finding of our study is that aortic cross-clamp and CPB durations were significantly shorter in the CAC group compared with the FAC group. In addition, groin-specific complications such as lymphatic leakage occurred in 6.5% of patients in the femoral cannulation group. No significant differences were observed between groups for mortality, intensive care unit stay, or neurological events. Aortic cross-clamp time was shorter in the CAC group (*p* = 0.015); however, after adjustment for baseline differences, cannulation strategy was not independently associated with cross-clamp duration, whereas it remained independently associated with CPB duration. These findings suggest that, in the right thoracotomy approach, CAC represents a safe and physiological alternative that avoids the peripheral vascular risks associated with femoral cannulation and may offer advantages in operative efficiency in selected patients.

In MICS procedures, FAC has long been accepted as a standard approach due to its ease of peripheral access and its lack of interference with the surgical field. However, the literature has reported that retrograde arterial perfusion is associated with an increased risk of stroke and neurological complications, particularly in patients with a high atherosclerotic burden [13].

Grossi et al. demonstrated that retrograde perfusion significantly increased the risk of stroke in high-risk patients with peripheral vascular disease or aortic pathology (*p* = 0.04; OR = 8.5) and emphasized that central cannulation should be preferred in such cases [10]. Similarly, Murzi and Glauber highlighted the “non-physiological” nature of retrograde perfusion and stated that central cannulation, by providing antegrade flow, reduces the risk of plaque embolization and iatrogenic aortic dissection [13].

Although no statistically significant difference in vascular embolic events was observed between groups in our study (CAC 1.1%, FAC 0%, *p* = 1.000), the physiological advantage of antegrade flow with central cannulation has been shown in the literature to confer a safer profile for cerebral protection [20,21].

Petersen et al. similarly reported that selectively applied antegrade axillary artery perfusion in patients with systemic atherosclerosis resulted in comparable in-hospital outcomes and similar stroke rates to those observed with retrograde femoral perfusion used in lower-risk patients. They argued that antegrade axillary perfusion constitutes a safe, minimally invasive treatment option in patients with higher preoperative risk and severe atherosclerosis. Cross-clamp times were also shorter compared with femoral cannulation, supporting our findings [22].

One of the notable findings of our study was the shorter operative times observed in the CAC group. In the FAC group, median CPB duration was 137 min and cross-clamp time was 90 min, whereas in the CAC group these durations were 114 min and 70.5 min, respectively (*p* < 0.05). However, after adjustment for baseline differences, cannulation strategy remained independently associated only with CPB duration. In addition, the relatively small sample size of the present study limits the strength and generalizability of these conclusions, and the findings should therefore be interpreted with caution. These observations are consistent with the large case series reported by Sharma et al., which included 958 patients and demonstrated that central cannulation via right anterolateral thoracotomy is safe, reproducible, and may simplify the surgical procedure by eliminating the need for an additional groin incision [14].

Conversely, some studies in the literature have suggested that minimally invasive approaches—particularly central cannulation—may require longer operative times than sternotomy or peripheral cannulation due to technical challenges [23,24]. However, our data and those of other studies indicate that, in experienced hands and with appropriate exposure techniques, central cannulation may shorten total operative time by eliminating the time required for peripheral vessel preparation and repair [14].

Furthermore, modified techniques reported by Liao and Zhang [25] and fully video-assisted thoracoscopic approaches described by Liu et al. [26] have demonstrated that the technical challenges of central cannulation can be overcome. These approaches highlight that central cannulation may be a life-saving alternative for patients with contraindications to peripheral cannulation, such as mural thrombus.

One of the principal goals of MICS is to reduce surgical trauma and procedure-related morbidity. Because femoral cannulation requires an additional incision, it carries the risk of local complications such as lymphocele, seroma, wound infection, and vascular injury [27]. In our study, lymphatic leakage was observed in 6.5% of patients in the FAC group, whereas it was naturally absent in the CAC group (*p* = 0.009), representing one of the most concrete advantages of the central approach. Abud et al. reported that central cannulation eliminated peripheral vascular complications and groin infections in obese patients [21]. Similarly, Flinspach et al. noted that peripheral cannulation—particularly when the internal jugular vein is used—may increase the risk of thrombosis [28]. However, because the number of observed events in our cohort was relatively small, these findings should be interpreted with caution and may not be fully generalizable.

Although no deep vein thrombosis was observed in the femoral cannulation group in our study, lymphatic complications resulting from surgical manipulation in the groin may adversely affect the recovery process. Central cannulation allows all procedures to be performed through a single thoracotomy incision, thereby adhering to the “single-incision” principle and eliminating groin-related morbidity. Beyond these surgical access–related considerations, the presence of peripheral vascular devices may represent another important factor influencing the choice between femoral and central cannulation strategies.

Femoral arterial cannulation may pose technical challenges, particularly in patients with stents and in those with prior endovascular interventions or peripheral vascular devices such as stents or grafts; moreover, the presence of inferior vena cava filters should be considered, as they may preclude venous cannulation in certain cases. Given that these devices carry risks of restenosis, thrombosis, vascular injury, endoleak, migration, fracture, and infection, central aortic cannulation—which avoids manipulation of peripheral vessels—may represent a practical alternative perfusion strategy in selected patients. Nevertheless, ongoing technological advances from conventional to functional stents may improve safety and durability, potentially allowing safer femoral arterial cannulation in selected patients when needed [29].

Recent advances in vascular device technology have also extended to inferior vena cava filters. A proof-of-concept study demonstrated that gadolinium nanoparticle–enhanced, bioresorbable poly(p-dioxanone)–based inferior vena cava filters improve radiopacity for fluoroscopic guidance and long-term CT monitoring without compromising clot-trapping efficacy or biocompatibility. Moreover, owing to their absorbable nature over time, these filters may reduce long-term complications associated with permanent devices and eliminate the need for retrieval procedures. Nevertheless, despite these technological advances, the presence of inferior vena cava filters may continue to complicate venous access strategies in selected patients and should therefore be considered during procedural planning. On the other hand, ongoing progress in device design and biomaterials may reduce the likelihood that central arterial or venous cannulation will be mandatory in the future [30].

In addition, the ability of bismuth nanoparticle–containing perivascular wraps to reduce neointimal hyperplasia may be noteworthy not only for arteriovenous fistula maturation but also for limiting peripheral vascular injury and supporting vessel repair processes that may be required after femoral cannulation. Given the potential for local vascular injury and the associated intimal response following femoral cannulation, biomaterial-based strategies that promote vascular healing may assume a complementary role in the future. This potential contribution may be particularly relevant in surgically challenging and complication-prone cases; however, further experimental and clinical studies are needed to clarify its clinical implications [31].

Regarding transfusion requirements, no significant differences were observed between groups in red blood cell or fresh-frozen plasma transfusions. However, platelet transfusion was marginally higher in the CAC group (*p* = 0.020). Although median platelet transfusion values were similar between groups, the overall distribution differed, with higher extreme values observed in the CAC group. This finding may reflect perioperative factors such as operative duration, bleeding profile, transfusion practices, and inflammatory response rather than the cannulation strategy itself. This observation may also be partly related to direct aortic cannulation; however, this remains speculative and cannot be confirmed in the present study. Previous studies have reported heterogeneous findings regarding transfusion requirements in minimally invasive cardiac surgery, with some analyses demonstrating reduced blood loss and lower transfusion requirements compared with conventional surgery. Meta-analytic data from other minimally invasive cardiac procedures have similarly reported reduced transfusion requirements despite longer operative times. Additional reports have demonstrated reduced blood product use and overall costs with central cannulation and lower transfusion rates in minimally invasive valve procedures compared with conventional surgery [21,32,33,34]. Although the difference in platelet use observed in our study does not suggest a major bleeding issue, it highlights the importance of meticulous hemostasis during central cannulation. Recent single-center experience has further supported the safety and feasibility of minimally invasive cardiac surgery across different stages of the learning curve, with favorable perioperative outcomes [4].

The present study has several limitations. First, its retrospective design inherently carries the risk of unmeasured confounding and limits causal inference. Second, this was a single-center experience based exclusively on in-hospital data, which represents an important limitation and may reduce the generalizability of the findings to other institutions with different patient populations, surgical expertise, and perioperative management practices. Third, the choice of cannulation strategy evolved over time according to surgical experience and clinical judgment rather than a predefined protocol; therefore, some degree of selection bias cannot be excluded. In addition, although baseline differences between groups were explored, comprehensive multivariable adjustment was limited, and residual confounding may persist. The relatively small sample size—particularly when patients were stratified according to different surgical procedures such as atrial septal defect repair and mitral valve surgery—may have reduced statistical power and limited the robustness of subgroup analyses. Because these procedures differ substantially in operative complexity, cardiopulmonary bypass duration, and cross-clamp time, part of the observed variability in operative outcomes may be attributable to procedural characteristics rather than solely to the cannulation strategy. Therefore, procedural heterogeneity should be considered when interpreting the results. Furthermore, because the analysis was restricted to in-hospital outcomes, the lack of long-term follow-up precluded assessment of late neurological events, vascular complications, and long-term survival. These limitations should be considered when interpreting the findings.

In terms of patient demographics, the CAC group in our study included a higher proportion of female patients (74.2%) and younger individuals, whereas patients in the FAC group were older and had a larger body surface area [23,26]. In our practice, the transition toward central arterial cannulation occurred during our institutional learning curve in minimally invasive cardiac surgery and was largely experience-driven, partly motivated by the intention to minimize potential risks associated with peripheral cannulation, particularly in patients with smaller body habitus—many of whom were female. As described in the Methods section, femoral arterial cannulation was routinely used during the early phase of the study period, whereas central aortic cannulation later became the preferred institutional strategy, with femoral access reserved for selected cases in which central cannulation was considered unsuitable based on preoperative imaging findings or intraoperative assessment. Accordingly, the observed demographic differences likely reflect changes in institutional practice over time rather than predefined anatomical selection criteria; however, this practice pattern may also represent a potential source of selection bias and should therefore be interpreted with caution.

Future prospective studies with extended follow-up are needed to better define the long-term safety profile of central versus femoral arterial cannulation, including late neurological and vascular events, functional recovery, and survival outcomes, which may further inform individualized patient selection and optimize procedural strategies in minimally invasive cardiac surgery.

## 5. Conclusions

This study suggests that CAC represents a feasible, safe, and effective alternative to FAC in minimally invasive cardiac surgery performed via right thoracotomy. Our data show that the central cannulation strategy was associated with shorter aortic cross-clamp and CPB durations. More importantly, groin incision–related morbidities such as lymphatic leakage were observed only in the femoral cannulation group in our cohort. Because central cannulation does not require an additional surgical incision and provides physiological antegrade flow, it may be considered, particularly in patients with unsuitable peripheral vascular anatomy or in whom femoral cannulation carries an increased risk. Although no significant differences were observed between the two techniques in terms of mortality or major neurological events in our study, the shorter operative times and absence of wound-related complications highlight the potential advantages of central cannulation for surgical efficiency and patient comfort. In experienced centers, CAC during right thoracotomy may represent a practical alternative approach while adhering to the fundamental principle of minimally invasive surgery—reducing surgical trauma. Future studies should evaluate the long-term outcomes and cost-effectiveness of this technique in larger and preferably prospective patient cohorts.

## Figures and Tables

**Table 1:** Baseline demographic and clinical characteristics according to arterial cannulation strategy.

Variable		Central Aortic Cannulation (*n* = 93)	Femoral Arterial Cannulation (*n* = 46)	*p* Value
Sex, *n* (%)	Female	69 (74.2)	21 (45.7)	0.001
	Male	24 (25.8)	25 (54.3)	
Age, years, median (IQR)		54.0 (25.0)	61.0 (21.0)	0.004
Height, m, mean ± SD		1.59 ± 0.09	1.65 ± 0.10	<0.001
Weight, kg, mean ± SD		72.4 ± 12.5	71.0 ± 9.2	0.482
Body surface area, m^2^, mean ± SD		1.7 ± 0.2	1.8 ± 0.2	0.043
Body mass index, kg/m^2^, mean ± SD		28.9 ± 5.2	27.2 ± 4.8	0.071
Hypertension, *n* (%)		38 (40.9)	9 (19.6)	0.013
Chronic obstructive pulmonary disease, *n* (%)		6 (6.5)	2 (4.3)	0.616
Cerebrovascular disease, *n* (%)		5 (5.4)	2 (4.3)	1.000
Diabetes mellitus, *n* (%)		14 (15.1)	8 (17.4)	0.722
Current smoking, *n* (%)		38 (40.9)	21 (45.7)	0.591
Pulmonary arterial hypertension, *n* (%)		7 (7.5)	3 (6.5)	0.829
Carotis stenosis, *n* (%)		16 (17.2)	4 (8.7)	0.179
Left ventricular ejection fraction, *n* (%)	<50%	13 (14.0)	7 (15.2)	0.845
	≥50%	80 (86.0)	39 (84.8)	
EuroSCORE category, *n* (%)	Low (≤3)	57 (61.3)	38 (78.3)	0.102
	Intermediate (4–6)	28 (30.1)	9 (19.6)	
	High (≥7)	8 (8.6)	1 (2.2)	

Data are presented as mean ± standard deviation or median (interquartile range), as appropriate. Abbreviations: EuroSCORE, European System for Cardiac Operative Risk Evaluation.

**Table 2:** Operative and early postoperative outcomes according to arterial cannulation strategy.

Variable	Central Aortic Cannulation (*n* = 93)		Femoral Arterial Cannulation (*n* = 46)		*p* Value
	Median (IQR)	Mean ± SD	Median (IQR)	Mean ± SD	
Aortic cross-clamp time, min, median (IQR)	70.5 (43.3)	74.8 ± 30.6	90.0 (38.3)	88.5 ± 29.2	0.015
Cardiopulmonary bypass duration, min, median (IQR)	114.0 (56.0)	124.1 ± 45.9	137.0 (91.8)	154.2 ± 56.7	0.003
Intensive care unit stay, days, median (IQR)	1.0 (1.0)	2.0 ± 2.1	1.0 (1.0)	1.9 ± 2.1	0.785
Hospital length of stay, days, median (IQR)	6.0 (2.0)	7.1 ± 3.1	6.0 (2.0)	7.9 ± 5.4	0.194
Red blood cell transfusion, units, median (IQR)	2.0 (2.0)	2.9 ± 3.1	2.0 (2.0)	2.3 ± 1.3	0.952
Fresh frozen plasma transfusion, units, median (IQR)	3.0 (2.0)	3.5 ± 2.6	3.0 (1.0)	2.9 ± 1.0	0.316
Platelet transfusion, units, median (IQR)	4.0 (1.0)	5.8 ± 5.3	4.0 (1.0)	4.5 ± 1.6	0.020
	*n*	%	*n*	%	
Postoperative deep vein thrombosis, *n* (%)	0 (0.0)	(0.0)	0 (0.0)	(0.0)	NA
Vascular embolic events, *n* (%)	1 (1.1)	(1.1)	0 (0.0)	(0.0)	1.000
In-hospital mortality, *n* (%)	6 (6.5)	(6.5)	0 (0.0)	(0.0)	0.188
Lymphatic leakage, *n* (%)	0 (0.0)	(0.0)	3 (6.5)	(6.5)	0.009

Data are presented as median (IQR) or number (percentage), as appropriate. Mean ± SD values were not used in statistical comparisons; they were provided for reference only. Abbreviations: NA, not applicable (no events occurred in either group).

**Table 3:** Perioperative laboratory parameters according to arterial cannulation strategy.

Variable	Time Point	Central Cannulation	Femoral Cannulation	*p* Value
Urea, mg/dL, median (IQR)	Preoperative	34.0 (19.5)	35.5 (16.5)	0.751
	Postoperative	35.0 (15.0)	35.0 (18.0)	0.597
Creatinine, mg/dL, median (IQR)	Preoperative	0.87 (0.28)	1.00 (0.33)	0.082
	Postoperative	0.80 (0.30)	0.84 (0.28)	0.255
AST, U/L, median (IQR)	Preoperative	19.0 (9.5)	22.0 (10.3)	0.093
	Postoperative	24.0 (13.0)	25.0 (22.5)	0.330
ALT, U/L, median (IQR)	Preoperative	16.0 (10.5)	18.0 (12.3)	0.265
	Postoperative	18.0 (20.8)	22.5 (20.0)	0.235
Neutrophil count, ×10^9^/L, median (IQR)	Preoperative	4.10 (2.40)	4.46 (2.56)	0.898
	Postoperative	5.59 (2.97)	5.80 (3.28)	0.665
Lymphocyte count, ×10^9^/L, median (IQR)	Preoperative	1.71 (0.82)	1.74 (0.89)	0.624
	Postoperative	1.68 (1.20)	1.55 (1.30)	0.728
Platelet count, ×10^9^/L, median (IQR)	Preoperative	232 (105)	243 (119)	0.954
	Postoperative	373 (0)	373 (0)	0.861

## Data Availability

Additional data are available upon reasonable request.

## Supplementary Materials

The following supporting information can be downloaded at: https://www.mdpi.com/article/10.3390/jcm15062383/s1, Table S1: Baseline demographic characteristics stratified by procedure type and cannulation strategy. Table S2: Intraoperative and early postoperative outcomes stratified by procedure type and arterial cannulation strategy. Table S3: Perioperative laboratory parameters stratified by procedure type and arterial cannulation strategy.

## Abbreviations

The following abbreviations are used in this manuscript:

CAC	Central arterial cannulation
CPB	Cardiopulmonary Bypass
FAC	Femoral arterial cannulation
MICS	Minimally invasive cardiac surgery
