# Supplementary material for: Central Aortic Cannulation in Minimally Invasive Cardiac Surgery via Right Thoracotomy: A Single-Center Retrospective Comparison
Source: J Clin Med. 2026 Mar 20;15(6):2383. doi: 10.3390/jcm15062383 (PMC13026934; doi:10.3390/jcm15062383)
Supplement: Supplementary file 1 [file jcm-15-02383-s001.zip › jcm-4191653-supplementary.pdf]

Supplementary Table S1. Baseline demographic characteristics stratified by procedure type and cannulation strategy

Variable		Overall(n = 139)	ASD (n = 26)Central (n = 16)	Femoral (n = 10)	*p* value	MVR+TRA (n = 33)Central (n = 22)	Femoral (n = 11)	*p* value	Isolated MVR (n = 51)Central (n = 35)	Femoral (n = 16)	*p* value	Other (n = 29)Central (n = 20)	Femoral (n = 9)	*p* value
Sex, n (%)	Male	49 (35.3)	6 (37.5)	7 (70.0)	0.107	2 (9.1)	4 (36.4)	0.146	8 (22.9)	9 (56.2)	**0.019**	8 (40.0)	5 (55.6)	0.688
	Female	90 (64.7)	10 (62.5)	3 (30.0)		20 (90.9)	7 (63.6)		27 (77.1)	7 (43.8)		12 (60.0)	4 (44.4)	
Age, years, mean ± SD		56.1 ± 15.5	29.1 ± 8.8	41.4 ± 14.8	**0.027**	56.9 ± 8.1	65.9 ± 7.4	**0.003**	56.4 ± 15.0	56.4 ± 13.5	0.990	52.7 ± 11.5	63.7 ± 12.1	**0.030**
Height, m, mean ± SD		1.61 ± 0.10	1.65 ± 0.10	1.70 ± 0.06	0.163	1.55 ± 0.08	1.59 ± 0.10	0.245	1.58 ± 0.08	1.66 ± 0.11	0.006	1.58 ± 0.09	1.65 ± 0.08	0.082
Weight, kg, mean ± SD		71.9 ± 11.5	72.4 ± 12.6	71.2 ± 9.9	0.798	70.5 ± 12.1	68.7 ± 11.0	0.685	73.9 ± 13.6	72.3 ± 7.6	0.661	71.8 ± 11.3	71.6 ± 9.8	0.968
Body surface area, m², mean ± SD		1.8 ± 0.2	1.8 ± 0.2	1.8 ± 0.1	0.633	1.7 ± 0.2	1.7 ± 0.2	0.464	1.7 ± 0.2	1.8 ± 0.2	0.173	1.7 ± 0.2	1.8 ± 0.2	0.214
Body mass index, kg/m², mean ± SD		28.3 ± 5.1	26.7 ± 4.7	24.5 ± 2.4	0.190	29.2 ± 4.1	28.7 ± 6.9	0.772	29.3 ± 5.5	27.0 ± 2.7	0.129	29.4 ± 5.9	28.7 ± 5.9	0.759
Hypertension, n (%)		47 (33.8)	2 (12.5)	1 (10.0)	1.000	10 (45.5)	2 (18.2)	0.125	16 (45.7)	4 (25.0)	0.160	10 (50.0)	2 (22.2)	0.160
COPD, n (%)		8 (5.8)	1 (6.3)	0 (0.0)	N/A	1 (4.5)	2 (18.2)	0.252	1 (2.9)	0 (0.0)	N/A	3 (15.0)	0 (0.0)	0.532
Cerebrovascular disease, n (%)		7 (5.0)	0 (0.0)	1 (10.0)	N/A	3 (13.6)	1 (9.1)	1.000	1 (2.9)	0 (0.0)	N/A	1 (5.0)	0 (0.0)	N/A
Diabetes mellitus, n (%)		22 (15.8)	1 (6.3)	1 (10.0)	1.000	4 (18.2)	1 (9.1)	0.643	6 (17.1)	2 (12.5)	1.000	3 (15.0)	4 (44.4)	0.158
Current smoking, n (%)		59 (42.4)	7 (43.8)	6 (60.0)	0.420	8 (36.4)	4 (36.4)	1.000	16 (45.7)	6 (37.5)	0.583	7 (35.0)	5 (55.6)	0.298
Peripheral Arterial Disease, n (%)		10 (7.2)	0 (0.0)	0 (0.0)	N/A	2 (9.1)	1 (9.1)	1.000	2 (5.7)	1 (6.3)	1.000	3 (15.0)	1 (11.1)	1.000
Carotis Stenosis, n (%)		20 (14.4)	1 (6.3)	0 (0.0)	N/A	3 (13.6)	0 (0.0)	0.534	6 (17.1)	2 (12.5)	0.672	6 (30.0)	2 (22.2)	0.665
Left ventricular ejection fraction, n (%)	<50%	20 (14.4)	0 (0.0)	0 (0.0)	N/A	6 (27.3)	2 (18.2)	0.566	4 (11.4)	2 (12.5)	1.000	3 (15.0)	3 (33.3)	0.339
	≥50%	119 (85.6)	16 (100)	10 (100)		16 (72.7)	9 (81.8)		31 (88.6)	14 (87.5)		17 (85.0)	6 (66.7)	
EuroSCORE category, n (%)	Low (≤3)	93 (66.9)	15 (93.8)	10 (100)	1.000	9 (40.9)	9 (81.8)	0.052	24 (68.6)	13 (81.3)	0.542	9 (45.0)	4 (44.4)	0.167
	Intermediate (4–6)	37 (26.6)	1 (6.3)	0 (0.0)		11 (50.0)	2 (18.2)		9 (25.7)	2 (12.5)		7 (35.0)	5 (55.6)	
	High (≥7)	9 (6.5)	0 (0.0)	0 (0.0)		2 (9.1)	0 (0.0)		2 (5.7)	1 (6.3)		4 (20.0)	0 (0.0)	

Data are presented as mean ± SD or number (percentage), as appropriate.

Supplementary Table S2. Intraoperative and early postoperative outcomes stratified by procedure type and arterial cannulation strategy

Variable	Overall (n=139)	ASD (n = 26)			MVR+TRA (n = 33)			Isolated MVR (n = 51)			Other procedures (n = 29)		
		Central (n=16)	Femoral (n=10)	p value	Central (n=22)	Femoral (n=11)	p value	Central (n=35)	Femoral (n=16)	p value	Central (n=20)	Femoral (n=9)	p value
**Aortic cross-clamp time, min**	76.0 (42.5)	47.5 (19.8)	46.5 (27.5)	0.452	63.0 (38.3)	101.0 (22.0)	<0.001	79.0 (38.0)	89.0 (28.0)	0.111	81.0 (63.5)	96.0 (65.0)	0.633
**Cardiopulmonary bypass duration, min**	124.0 (67.0)	94.5 (44.3)	94.0 (29.3)	0.623	135.0 (48.8)	215.0 (62.0)	<0.001	119.0 (57.0)	126.0 (52.8)	0.212	103.5 (87.3)	144.0 (94.0)	0.105
**ICU stay, days**	1.0 (1.0)	1.0 (0.0)	1.0 (0.0)	1.000	1.0 (1.0)	2.0 (1.0)	0.510	1.0 (1.0)	1.0 (1.0)	0.801	2.0 (3.8)	2.0 (3.5)	0.660
**Hospital stay, days**	6.0 (2.5)	5.0 (0.0)	5.0 (2.5)	0.220	6.0 (3.5)	6.0 (1.0)	0.785	6.0 (1.0)	7.0 (2.0)	0.090	7.0 (7.0)	7.0 (4.0)	0.629
**Erythrocyte suspension units**	2.0 (2.0)	1.0 (0.0)	0.0 (0.0)	0.769	2.0 (2.0)	1.0 (1.5)	0.530	2.5 (2.0)	2.0 (3.0)	0.901	3.0 (4.0)	3.0 (2.0)	1.000
**Fresh frozen plasma units**	3.0 (1.3)	2.0 (1.0)	2.0 (1.0)	0.897	3.0 (2.0)	3.0 (1.0)	0.534	3.0 (2.0)	2.5 (1.0)	0.275	3.0 (3.0)	3.0 (1.0)	0.908
**Platelet units**	4.0 (1.0)	4.0 (1.0)	4.0 (0.0)	0.623	4.0 (0.3)	4.0 (0.0)	0.534	5.0 (1.0)	4.0 (1.0)	0.139	5.0 (4.0)	5.0 (1.0)	0.153
**Postoperative deep vein thrombosis, n (%)**	0 (0.0)	0 (0.0)	0 (0.0)	N/A	0 (0.0)	0 (0.0)	N/A	0 (0.0)	1 (2.9)	N/A	0 (0.0)	0 (0.0)	N/A
**Vascular embolic events, n (%)**	1 (0.7)	0 (0.0)	0 (0.0)	N/A	0 (0.0)	0 (0.0)	N/A	0 (0.0)	0 (0.0)	N/A	0 (0.0)	0 (0.0)	N/A
**In-hospital mortality, n (%)**	6 (4.3)	0 (0.0)	0 (0.0)	N/A	1 (4.5)	0 (0.0)	N/A	4 (11.4)	0 (0.0)	0.394	1 (5.0)	0 (0.0)	N/A
**Lymphatic leakage, n (%)**	3 (2.2)	0 (0.0)	0 (0.0)	N/A	0 (0.0)	2 (18.2)	N/A	0 (0.0)	0 (0.0)	N/A	0 (0.0)	1 (11.1)	N/A

Data are presented as mean ± SD or number (percentage), as appropriate.

Supplementary Table S3. Perioperative laboratory parameters stratified by procedure type and arterial cannulation strategy

Variable	Time point	Overall (n=139)	ASD (n = 26)			MVR+TRA (n = 33)			Isolated MVR (n = 51)			Other procedures (n = 29)		
			Central (n=16)	Femoral (n=10)	p value	Central (n=22)	Femoral (n=11)	p value	Central (n=35)	Femoral (n=16)	p value	Central (n=20)	Femoral (n=9)	p value
**Urea, mg/dL**	Preoperative	35.0 (18.0)	26.5 (14.3)	36.0 (9.5)	0.121	43.0 (29.8)	35.0 (14.0)	0.143	32.0 (18.0)	34.0 (23.5)	0.823	39.5 (32.8)	45.0 (44.5)	0.908
	Postoperative	35.0 (16.5)	35.5 (20.8)	30.5 (12.8)	0.979	35.5 (15.8)	36.0 (17.0)	0.560	34.5 (14.3)	36.5 (38.0)	0.876	39.0 (18.0)	39.0 (19.5)	0.764
**Creatinine, mg/dL**	Preoperative	0.90 (0.28)	0.78 (0.24)	0.93 (0.21)	0.068	0.87 (0.46)	0.91 (0.26)	0.778	0.89 (0.27)	1.03 (0.36)	0.146	0.94 (0.34)	1.17 (0.77)	0.908
	Postoperative	0.82 (0.29)	0.73 (0.28)	0.74 (0.27)	0.979	0.82 (0.33)	0.86 (0.33)	0.462	0.79 (0.30)	0.86 (0.34)	0.126	0.89 (0.31)	0.82 (0.46)	0.982
**AST, U/L**	Preoperative	20.0 (9.0)	17.5 (6.8)	18.5 (6.3)	0.201	18.5 (10.5)	24.0 (5.0)	0.105	20.0 (8.0)	20.5 (5.8)	0.753	24.0 (20.5)	30.0 (10.0)	0.274
	Postoperative	25.0 (17.0)	18.0 (4.5)	26.0 (15.0)	0.005	26.0 (10.3)	25.0 (27.0)	0.693	24.5 (16.3)	19.0 (18.5)	0.349	30.5 (14.8)	36.0 (27.5)	0.417
**ALT, U/L**	Preoperative	17.0 (10.0)	16.0 (7.0)	16.0 (7.8)	0.452	14.0 (11.0)	18.0 (9.0)	0.299	19.0 (16.0)	16.0 (16.0)	0.428	17.5 (29.5)	31.0 (19.5)	0.095
	Postoperative	20.0 (20.0)	17.0 (16.0)	19.5 (14.0)	0.262	15.0 (16.5)	24.0 (20.0)	0.218	26.5 (25.0)	24.5 (19.8)	0.811	17.5 (20.5)	20.0 (39.0)	0.627
**Neutrophil count, $\times 10^9$/L**	Preoperative	4.11 (2.44)	3.70 (2.39)	3.25 (2.88)	0.897	4.28 (2.19)	4.70 (2.60)	0.462	4.32 (2.68)	4.43 (2.35)	0.768	3.51 (2.75)	4.42 (4.90)	1.000
	Postoperative	5.69 (3.20)	4.70 (2.80)	4.25 (2.93)	0.856	5.59 (4.29)	6.10 (5.10)	0.281	6.17 (2.29)	5.95 (2.43)	0.942	5.21 (3.08)	5.44 (5.00)	0.908
**Lymphocyte count, $\times 10^9$/L**	Preoperative	1.71 (0.82)	1.90 (1.23)	1.90 (0.54)	0.776	1.61 (1.39)	1.20 (1.10)	0.336	1.71 (0.58)	1.92 (1.05)	0.098	1.50 (0.98)	1.46 (0.58)	0.729
	Postoperative	1.67 (1.21)	1.69 (1.23)	2.00 (1.33)	0.737	1.59 (1.38)	1.21 (0.50)	0.440	1.71 (0.68)	1.90 (1.44)	0.411	1.25 (1.45)	1.69 (1.38)	0.472
**Platelet count, $\times 10^9$/L**	Preoperative	234.0 (106.5)	243.0 (87.0)	259.0 (69.5)	0.598	245.5 (113.8)	163.0 (47.0)	0.040	217.0 (89.3)	243.5 (115.5)	0.170	245.5 (101.8)	248.0 (152.5)	0.871
	Postoperative	213.5 (102.8)	216.0 (135.3)	222.0 (101.5)	0.760	198.5 (84.0)	184.0 (110.0)	0.665	215.0 (103.0)	216.0 (60.3)	0.941	188.0 (176.5)	267.0 (116.5)	0.216

Data are presented as mean ± SD or number (percentage), as appropriate.
